# Supplementary material for: Differential Effects of Exercise on fMRI of the Midbrain Ascending Arousal Network Nuclei in Myalgic Encephalomyelitis/Chronic Fatigue Syndrome (ME/CFS) and Gulf War Illness (GWI) in a Model of Postexertional Malaise (PEM)
Source: Brain Sci. 2022 Jan 5;12(1):78. doi: 10.3390/brainsci12010078 (PMC8774249; doi:10.3390/brainsci12010078)
Supplement: Supplementary file 1 [file brainsci-12-00078-s001.zip › brainsci-1497146-supplementary.pdf]

brain sciences

Supplementary On-line Material

Differential Effects of Exercise on fMRI of the Midbrain Ascending Arousal Network Nuclei in Myalgic Encephalomyelitis / Chronic Fatigue Syndrome (ME/CFS) and Gulf War Illness (GWI) in a Model of Postexertional Malaise

James N. Baraniuk MD, Alison Amar MS, Haris Pepermitwala MS, Stuart Washington PhD

Running title: Midbrain in PEM, ME/CFS and GWI

BACKGROUND for Figure S1: BOLD of the original region of interest (ROI).

In a previous study [14] we examined blood oxygenation level dependent (BOLD) activation during a difficult, high cognitive load 2-back task and compared preexercise and postexercise scans. Control, ME/CFS and GWI were equivalent prior to exercise (baseline), but after exercise ME/CFS had a significant increase in BOLD while GWI had a significant decrease in the dorsal midbrain, right middle insula and left Rolandic operculum [14]. The midbrain region of interest extended from the left to right periaqueductal gray (PAG) and to the adjacent right midbrain reticular formation (MRF), inferior colliculus and lateral lemniscus, and caudally to the right lateral isthmus (Figure S1). Because these nuclei have profound influences on threat assessment, pain, negative emotion, attention, wakefulness, and instinctual neurobehaviours, it was of interest to assess the activation of relevant anatomical midbrain nuclei.

METHOD:

The original region of interest [14] was portrayed with the ascending arousal network [36,37] on slices in the x, y and z planes.

RESULTS for Figure S1:

The seed region approach identified significant differences in BOLD in nuclei that appeared to be outside the region of interest found in the original study (Figure S1). This is possible because > 35 contiguous voxels were required for the ROI analysis and the midbrain activation may have been patchy in the smaller nuclei. It was noted that before exercise ME/CFS had significantly lower BOLD compared to control and GWI when all ascending arousal network nodes were compared. Similarly, after exercise GWI had significantly reduced BOLD compared to ME/CFS. Explanations include exercise-induced changes in cerebral blood flow or neurovascular coupling. Although the same nuclei were impacted, the mechanisms may be unique to each disease.

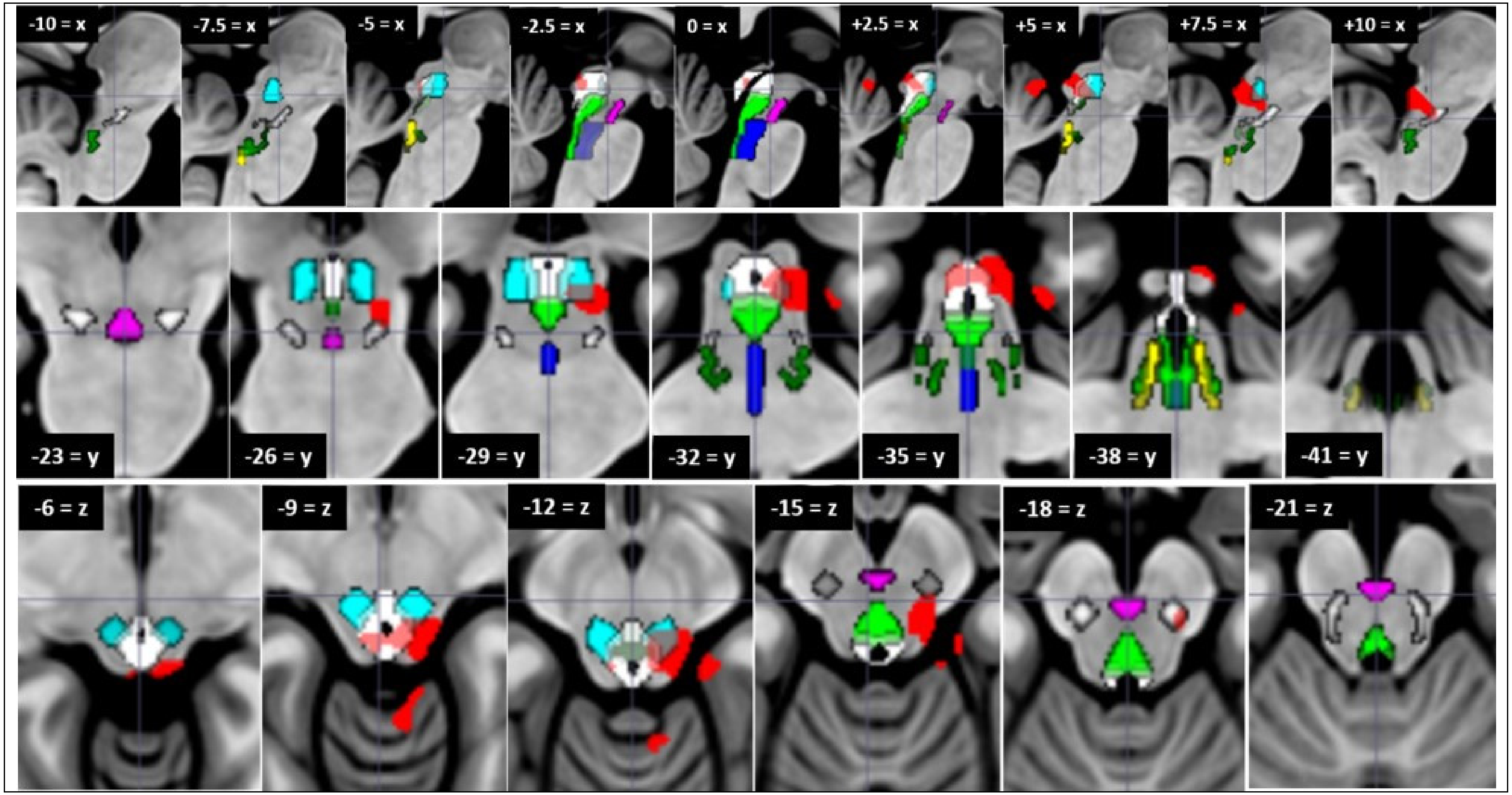

Figure S1. Midbrain region of interest. The 141 voxel (red) ROI [14] intersected nuclei of the Ascending Arousal Network in the x, y, z planes (MNI coordinate system). In the sagittal slices of the x plane, the ROI extended from the left periaquaductal grey (PAG, white, x = -2.5) through the right midbrain reticular formation (cyan) to the edge of the right pedunculotegmental nucleus (formerly pontopeduncular nucleus, PPN, grey, x=10) and lateral lemniscus at the right lateral margin of the dorsal midbrain (edge at x=12.5). Coronal slices (y plane) showed the right-sided ROI in the region of the inferior colliculus (y = -35), PAG (white), midbrain reticular formation (cyan, y = -29), and cuneiform nucleus (y = -26). Axial slices (z plane) outlined the inferior colliculus (z = -6), PAG (white), midbrain reticular formation (cyan) and region lateral to the dorsal raphe (lime green, z = -15) extending towards the right pedunculotegmental nucleus (grey, z = -15). The ROI was bounded on the rostral side by the superior colliculus, in the ventral midbrain by dorsal raphe (lime green), median raphe (blue), and ventral tegmental area (magenta), and on the caudal end by nuclei in the isthmus of the rostral hindbrain including locus coeruleus (yellow) and adjacent parabrachial complex (dark green) (y = -38). The 141 voxels in the ROI (red) were contiguous. Additional regions with fewer than 35 contiguous voxels that corresponded to

other nuclei of the ascending arousal network may have also been activated and significant by the seed region approach but would have not been to small to be significant in the original ROI analysis [14].

METHOD for multivariate general linear modeling of demographics and questionnaire data and Tables S1 and S2.

Preexercise BOLD values were assessed by self-reported demographics variables in multivariable general linear model: Age + gender + PTSD + BMI + Dolorimetry (kg) + Disease status (1 HC, 2 CFS, 3 GWI) + Orthostatic status (1 POTS, 2 START, 3 STOPP) + Marital status (1 single, 2, living together, 3 married, 4 divorced or separated, 5 widow widower) + Home assistance (1 caretaker) + Work status (1 Unemployed, 2 student, 3 Homemaker, 4 retired in good health, 5 Disabled but working, 6 Disabled) + Pain Treatment (0 none,_1 once per wk, 2 1 or 2 days per week, 3 more than 2 days per week, 4 daily) + Smoking (0 Never, 1 quit 6 months ago, 2_Yes, still smoking) + Fibromyalgia + Chronic Fatigue Syndrome + Allergic Rhinitis + Sinusitis + Nasal Polyps + Asthma + Depression + Diabetes + Thyroid disease + Bronchitis Emphysema or COPD + Heart disease + High blood pressure + Stroke + Acid reflux ulcers other stomach or intestinal problem + Liver disease + Kidney disease + Back pain Neck + Back pain Middle Back + Back pain Low back + Osteoarthritis degenerative arthritis + Rheumatoid arthritis or other autoimmune disease + Anemia other blood problem or disease + Cancer. The significant covariates were Orthostatic status, low back pain, depression, heart disease, gender and marital status (Table S1).

The next iteration used the significant covariates as fixed factors and removed the other variables. Orthostatic status was the only variable to be significant (Table S2) and was investigated in the other models.

Table S1. Preexercise multivariate general linear modal for BOLD with self-reported demographics as independent variables.

Tests of Between-Subjects Effects	Partial Eta Squared	Orthostatic status	Low back pain	CFS	Depression	Heart disease	Gender	Marital status
DR	0.048					0.026		
L_MRF	0.04		0.042		0.037			
L_PBC	0.058	0.014						
L_PO	0.091						0.002	0.003
L_PO	0.085							
L_PPN	0.058							0.014
PAG	0.058					0.015		
R_LC	0.041							0.041
R_PBC	0.056							0.016
R_PO	0.044						0.033	0.026
R_PPN	0.064			0.01				
VTA	0.042						0.038	
Pillai's Trace		0.227	0.239	0.281		0.304	0.25	0.258
F		1.844	1.971	2.462		2.748	2.093	2.182

Hypothesis df		14	14	14		14	14	14
Error df		88	88	88		88	88	88
Sig.		0.044	0.029	0.006		0.002	0.02	0.014
Partial Eta Squared		0.227	0.239	0.281		0.304	0.25	0.258

Table S2. Preexercise mGLM of demographic fixed factors. Orthostatic status, Low back pain (LBP), Depression, Heart disease, gender and Marital status were fixed factors with no other covariates.

Multivariate Tests	Effect	Value	F	Hypothesis df	Error df	Sig.	Partial Eta Squared
Orthostatic status	Roy's Largest Root	0.391	1.982	14	71	0.032	0.281
Gender * Marital	Roy's Largest Root	0.414	2.100	14	71	0.022	0.293
LBP * Marital	Roy's Largest Root	0.442	2.239	14	71	0.014	0.306
LBP * Gender * Orthostatic status	Pillai's Trace	0.498	1.681	28	142	0.026	0.249

METHOD for Partial correlations and Tables S3 to S6

Partial correlations compared BOLD signal intensities for each node on Day 1, Day 2 and the delta with subjective questionnaires about CFS symptoms, SF36 domains, psychological and depression complaints with disease status, orthostatic status, gender, age and BMI as covariates.

RESULTS: for Partial correlations and Tables S3 to S6

BOLD data were internally correlated within Day 1, Day 2 and delta, positively correlated between Day 2 and delta, and negatively correlated for Day 1 vs delta (Tables S3-S6). There were no significant correlations between subjective questionnaire data and objective preexercise or postexercise BOLD outcomes. The magnitudes of the significant correlations ($p < 0.05$ corrected) were low ($R > 0.4$, $R < -0.4$ for the inversely scored SF36 domains).

Table S3. Partial correlations between BOLD and questionnaire data. Partial correlations were assessed with disease status, orthostatic status, gender, age, PTSD, tenderness by dolorimetry and BMI as covariates. The upper left corner shows correlations for BOLD signal in each node from Day1, Day 2 (cyan edge) and Delta BOLD. BOLD was highly correlated within each day. Delta BOLD was positively correlated with Day 2 and negatively correlated with Day 1. Preexercise was not correlated with postexercise indicating that the exercise protocol induced alterations of the correlation structure. Symptoms were not correlated with BOLD measurements. SF-36 quality of life scores were negatively correlated with the other subjective questionnaires. Nodes and questionnaire items were color coded and are listed below in Tables S2 to S4. Correlations with R > 0.4 (R < -0.4) were significant (p < 0.05 uncorrected).

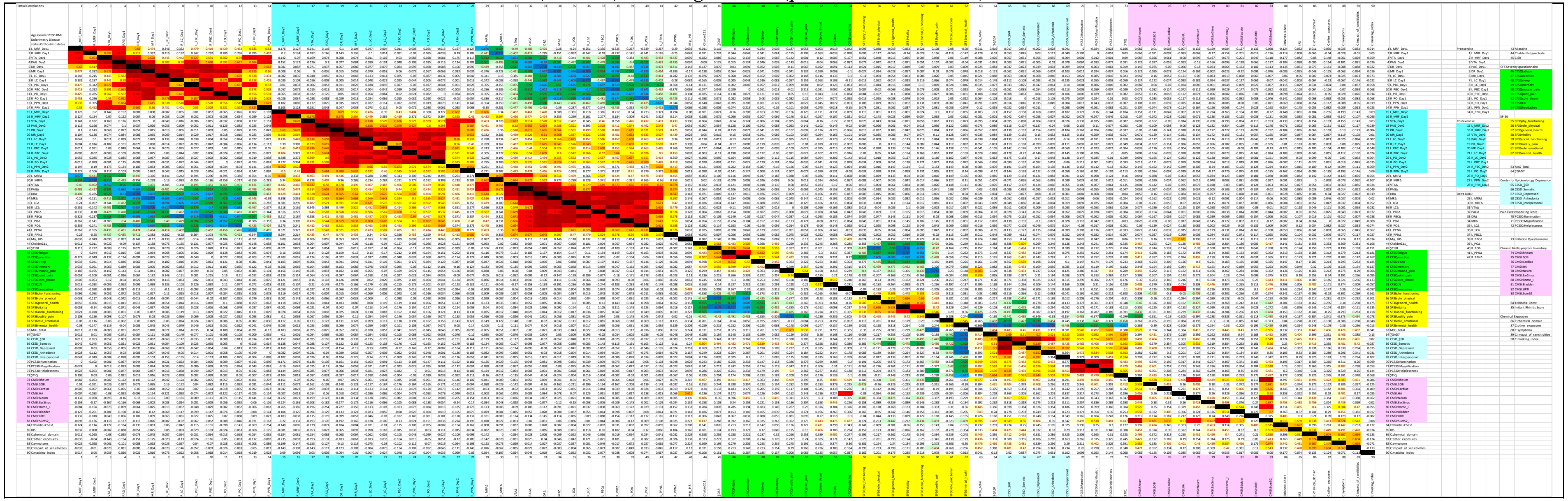

Table S4. Legend for significance of partial correlations for Table S3.

Color	R
	1
	> 0.9
	> 0.8
	> 0.7
	> 0.6
	> 0.5
	> 0.4
	< -0.4
	< -0.5
	< -0.6
	< -0.7
	< -0.8

Table S5. Partial correlations for Preexercise, Postexercise and Delta BOLD in the upper left corner of Table S3.

Preexercise		Postexercise		Delta BOLD	
1	L_MRF_Day1	15	L_MRF_Day2	29	L_MRFΔ
2	R_MRF_Day1	16	R_MRF_Day2	30	R_MRFΔ
3	VTA_Day1	17	VTA_Day2	31	VTAΔ
4	PAG_Day1	18	PAG_Day2	32	PAGΔ
5	DR_Day1	19	DR_Day2	33	DRΔ
6	MR_Day1	20	MR_Day2	34	MRΔ
7	L_LC_Day1	21	L_LC_Day2	35	L_LCΔ
8	R_LC_Day1	22	R_LC_Day2	36	R_LCΔ
9	L_PBC_Day1	23	L_PBC_Day2	37	L_PBCΔ
10	R_PBC_Day1	24	R_PBC_Day2	38	R_PBCΔ
11	L_PO_Day1	25	L_PO_Day2	39	L_POΔ
12	R_PO_Day1	26	R_PO_Day2	40	R_POΔ
13	L_PPN_Day1	27	L_PPN_Day2	41	L_PPNΔ
14	R_PPN_Day1	28	R_PPN_Day2	42	R_PPNΔ

Table S6. Partial correlations for subjective symptom severities in the lower right corner of Table S3.

Row Column	Item		
43	Migraine	Pain Catastrophizing Score	
44	Chalder Fatigue Scale	70	PCS30:Rumination
45	CISR	71	PCS30:Magnification
		72	PCS30:Helplessness
CFS Severity questionnaire			
46	CFSQfatigue	73	Irritation Questionnaire
47	CFSQexertion		
48	CFSQsleep	Chronic Multisymptom Inventory	
49	CFSQmemory	74	CMSI:Rheum
50	CFSQmuscle_pain	75	CMSI:SOB
51	CFSQjoint_pain	76	CMSI:Cardiac
52	CFSQsore_throat	77	CMSI:HA
53	CFSQLN	78	CMSI:Neuro
54	CFSQheadaches	79	CMSI:EarSinus
		80	CMSI:Rome_I
SF-36		81	CMSI:Bladder
55	SF36phy_functioning	82	CMSI:URTI
56	SF36role_physical	83	CMSI:Sum
57	SF36general_health		
58	SF36vitality	84	ΣRhinitis+Chest
59	SF36social_functioning	85	Irritant Rhinitis Score
60	SF36bodily_pain		
61	SF36role_emotional	Chemical Exposures	
62	SF36mental_health	86	C:chemical_domain
		87	C:other_exposures
63	McGill Pain Total	88	C:symptoms
64	General Anxiety GAD7	89	C:impact_of_sensitivities
		90	C:masking_index
Center for Epidemiology Depression			
65	CESD_∑60		
66	CESD_Somatic		
67	CESD_Depressed		
68	CESD_Anhedonia		
69	CESD_Interpersonal		

METHOD for Preexercise analysis.

Data were parsed by Disease status and Orthostatic status. Comparisons were made by ANOVA and Tukey Honest Significant Difference and multivariate general linear modeling (SPSS27).

RESULTS for Preexercise analysis. Tables S7 and S8.

Orthostatic status preexercise. The only significant difference was POTS greater than STOPP for L_PBC (mean ± 95%CI, Tukey Honest Significant Difference) (Table S7).

Gender status preexercise found significantly larger. L_PO in males than females. There was a general trend for greater BOLD in males than females Mean ± 95%CI (Table S8).

Table S7. Orthostatic status preexercise. The only significant difference was POTS greater than STOPP for L_PBC (mean ± 95%CI, Tukey Honest Significant Difference).

	POTS	START	STOPP	POTS>STOPP
N	21	40	84	
L_MRF	0.181 ± 0.139	0.241 ± 0.105	0.174 ± 0.105	
R_MRF	0.201 ± 0.175	0.220 ± 0.100	0.220 ± 0.100	
PAG	0.193 ± 0.188	0.239 ± 0.085	0.157 ± 0.085	
VTA	0.199 ± 0.124	0.212 ± 0.068	0.175 ± 0.068	
DR	0.258 ± 0.162	0.268 ± 0.079	0.163 ± 0.079	
MR	0.328 ± 0.163	0.308 ± 0.083	0.167 ± 0.083	
L_PO	0.308 ± 0.165	0.257 ± 0.083	0.162 ± 0.083	
R_PO	0.260 ± 0.147	0.314 ± 0.090	0.171 ± 0.090	
L_PPN	0.265 ± 0.147	0.205 ± 0.084	0.219 ± 0.084	
R_PPN	0.328 ± 0.152	0.261 ± 0.073	0.219 ± 0.073	
L_LC	0.368 ± 0.193	0.292 ± 0.103	0.154 ± 0.103	
R_LC	0.355 ± 0.196	0.393 ± 0.120	0.156 ± 0.120	
L_PBC	0.380 ± 0.197	0.273 ± 0.091	0.146 ± 0.091	0.046
R_PBC	0.242 ± 0.171	0.308 ± 0.099	0.185 ± 0.099	

Table S8. Gender status preexercise. L_PO was significantly larger in males than females. There was a general trend for greater BOLD in males than females. Mean ± 95%CI.

	Female	Male	Male > Female
N	54	91	
L_MRF	0.158 ± 0.118	0.215 ± 0.091	
R_MRF	0.208 ± 0.113	0.222 ± 0.096	
PAG	0.158 ± 0.104	0.201 ± 0.079	
VTA	0.149 ± 0.078	0.213 ± 0.060	
DR	0.156 ± 0.091	0.235 ± 0.073	
MR	0.177 ± 0.099	0.260 ± 0.079	
L_PO	0.116 ± 0.099	0.265 ± 0.074	0.017
R_PO	0.143 ± 0.107	0.270 ± 0.080	

L_PPN	0.215 ± 0.095	0.226 ± 0.080	
R_PPN	0.220 ± 0.075	0.262 ± 0.077	
L_LC	0.139 ± 0.123	0.273 ± 0.092	
R_LC	0.186 ± 0.146	0.289 ± 0.112	
L_PBC	0.154 ± 0.110	0.250 ± 0.085	
R_PBC	0.155 ± 0.123	0.270 ± 0.088	

METHOD for Postexercise analysis. Tables S9 and 10.

Data were parsed by Disease status and Orthostatic status. Comparisons were made by ANOVA and Tukey Honest Significant Difference and multivariate general linear modeling (SPSS27).

RESULTS for Postxercise analysis. Tables S9 and 10.

Orthostatic status postxercise. There were no significant differences based on Orthostatic status following exercise. Mean ± 95%CI. Tukey Honest Significant Difference (Table S9).

Gender status postexercise. There was no differences between women and men following exercise (Table S10)

Table S9. Postexercise orthostatic status.

	STOPP	START	POTS
	84	40	21
L_MRF	0.165 ± 0.090	0.200 ± 0.151	0.088 ± 0.184
R_MRF	0.209 ± 0.089	0.204 ± 0.139	0.115 ± 0.152
PAG	0.139 ± 0.081	0.190 ± 0.143	0.041 ± 0.123
VTA	0.161 ± 0.067	0.147 ± 0.104	0.071 ± 0.112
DR	0.161 ± 0.072	0.174 ± 0.123	0.068 ± 0.118
MR	0.182 ± 0.075	0.191 ± 0.130	0.079 ± 0.140
L_PO	0.203 ± 0.082	0.125 ± 0.117	0.103 ± 0.165
R_PO	0.209 ± 0.077	0.124 ± 0.124	0.110 ± 0.161
L_PPN	0.150 ± 0.076	0.214 ± 0.129	0.038 ± 0.163
R_PPN	0.191 ± 0.077	0.235 ± 0.125	0.206 ± 0.139
L_LC	0.204 ± 0.091	0.150 ± 0.128	0.057 ± 0.177
R_LC	0.202 ± 0.099	0.144 ± 0.150	0.148 ± 0.166
L_PBC	0.206 ± 0.084	0.172 ± 0.123	0.040 ± 0.211
R_PBC	0.223 ± 0.079	0.122 ± 0.119	0.136 ± 0.169

Table S10. Postexercise gender status.

	Male	Female
	91	54
L_MRF	0.203 ± 0.083	0.097 ± 0.127
R_MRF	0.219 ± 0.085	0.152 ± 0.111
PAG	0.177 ± 0.075	0.074 ± 0.112

VTA	0.180 ± 0.059	0.084 ± 0.090
DR	0.178 ± 0.067	0.107 ± 0.098
MR	0.192 ± 0.072	0.132 ± 0.102
L_PO	0.183 ± 0.076	0.140 ± 0.105
R_PO	0.182 ± 0.072	0.153 ± 0.108
L_PPN	0.194 ± 0.077	0.079 ± 0.097
R_PPN	0.248 ± 0.070	0.135 ± 0.104
L_LC	0.188 ± 0.087	0.134 ± 0.109
R_LC	0.210 ± 0.088	0.124 ± 0.131
L_PBC	0.174 ± 0.081	0.170 ± 0.114
R_PBC	0.210 ± 0.072	0.136 ± 0.111

METHODS for Preexercise Multivariate general linear models (mGLM) and Tables S11 to S13.

Preexercise mGLM used Disease, Orthostatic, PTSD and gender as fixed factors with age, BMI and dolorimetry pressure thresholds as independent variables.

RESULTS for Preexercise Multivariate general linear models (mGLM) and Table S11 to S13.

Prior to exercise, Disease status was significant (Wilks' lambda = 0.662, p = 0.028, Partial Eta Squared = 0.187) (Table S11) with L_PTN having significantly lower BOLD in CFS (0.018 ± 0.143, mean ± 95%CI) than control (0.326 ± 0.198, p = 0.047 univariate significance) and GWI (0.286 ± 0.127, p = 0.018) (Table S12). This was comparable to the ANOVA outcomes (Table 3).

Orthostatic status was significant (Roy's largest root = 0.256, p = 0.041, Partial Eta Squared = 0.204) with L_PBC being significantly lower in STOPP (0.062 ± 0.121, mean ± 95%CI) than POTS (0.394 ± 0.219, p = 0.006 Tukey Honest Significant Difference) and START (0.397 ± 0.164, p = 0.034) (Table S13).

Age, gender, PTSD, BMI and dolorimetry pressure thresholds were not significant covariates prior to exercise.

Table S11. Preexercise model. Disease, Orthostatic, PTSD and gender were fixed factors with age, BMI and dolorimetry pressure thresholds as independent variables. Disease status (Wilks' Lambda = 0.662, p = 0.028, Partial Eta Squared = 0.187) and the Orthostatic status * Gender cross-product (Roy's Largest Root = 0.299, p = 0.014, Partial Eta Squared = 0.23) were significant in the model.

Tests of Between-Subjects Effects	Partial Eta Squared	Disease status	Orthostatic status	PTSD	Orthostatic status * Gender
L_PPN	0.109	0.001			
L_PBC	0.071		0.016		
R_LC	0.044			0.024	
R_MRF	0.061				0.028

Table S12. Disease status in preexercise mGLM. Estimated marginal means (mean ± 95%CI) for Disease status were significantly greater in control and GWI than CFS in L_PPN (univariate significance). The model contrasts with the differences by ANOVA in Table 3.

	Control	CFS	GWI	Control>CFS	GWI>CFS
L_MRF	0.327 ± 0.248	0.152 ± 0.179	0.182 ± 0.159		
R_MRF	0.461 ± 0.252	0.268 ± 0.182	0.128 ± 0.162		
PAG	0.256 ± 0.211	0.111 ± 0.152	0.232 ± 0.135		
VTA	0.314 ± 0.164	0.104 ± 0.119	0.146 ± 0.106		
DR	0.218 ± 0.191	0.120 ± 0.139	0.268 ± 0.123		
MR	0.205 ± 0.206	0.144 ± 0.149	0.314 ± 0.133		
L_PO	0.217 ± 0.193	0.056 ± 0.139	0.271 ± 0.124		
L_PPN	0.326 ± 0.198	0.018 ± 0.143	0.286 ± 0.127	0.047	0.018
R_PO	0.206 ± 0.214	0.116 ± 0.155	0.293 ± 0.137		
R_PPN	0.384 ± 0.188	0.132 ± 0.137	0.268 ± 0.121		
L_LC	0.160 ± 0.246	0.201 ± 0.178	0.320 ± 0.158		
R_LC	0.047 ± 0.297	0.169 ± 0.215	0.371 ± 0.191		

L_PBC	0.314 ± 0.213	0.177 ± 0.155	0.317 ± 0.137		
R_PBC	0.176 ± 0.242	0.130 ± 0.175	0.281 ± 0.156		

Table S13. Orthostatic status in preexercise mGLM. Estimated marginal means (mean ± 95%CI) for orthostatic status were significantly greater in POTS and START than STOPP in L_PBC (univariate significance).

	POTS	START	STOPP	POTS>STOPP	START>STOPP
L_MRF	0.098 ± 0.254	0.314 ± 0.190	0.210 ± 0.140		
R_MRF	0.154 ± 0.258	0.360 ± 0.193	0.255 ± 0.143		
PAG	0.104 ± 0.216	0.353 ± 0.161	0.136 ± 0.120		
VTA	0.162 ± 0.168	0.255 ± 0.126	0.125 ± 0.093		
DR	0.193 ± 0.196	0.320 ± 0.146	0.117 ± 0.108		
MR	0.267 ± 0.211	0.332 ± 0.158	0.110 ± 0.117		
L_PO	0.238 ± 0.198	0.264 ± 0.148	0.090 ± 0.110		
L_PPN	0.291 ± 0.202	0.242 ± 0.151	0.144 ± 0.112		
R_PO	0.198 ± 0.219	0.326 ± 0.164	0.125 ± 0.121		
R_PPN	0.288 ± 0.193	0.301 ± 0.144	0.203 ± 0.106		
L_LC	0.358 ± 0.252	0.307 ± 0.189	0.080 ± 0.139		
R_LC	0.255 ± 0.304	0.343 ± 0.227	0.071 ± 0.168		
L_PBC	0.394 ± 0.219	0.397 ± 0.164	0.062 ± 0.121	0.006	0.034
R_PBC	0.154 ± 0.248	0.329 ± 0.186	0.131 ± 0.138		

METHODS for Postexercise Multivariate general linear models (mGLM) and Tables S14 to S18.

Postexercise mGLM used Disease, Orthostatic, PTSD and gender as fixed factors with age, BMI and dolorimetry pressure thresholds as independent variables.

RESULTS for Postexercise Multivariate general linear models (mGLM) and Table S14 to S18.

The model was significant for Disease status postexercise (Wilks' lambda = 0.665, p = 0.033, Partial Eta Squared = 0.184). CFS and Control had significantly higher BOLD activation than GWI in L_MRF, VTA, and R_PPN (Table S14). CFS was greater than GWI for all except L_PO, L_LC and L_PBC. The mGLM identified more significant nodes than ANOVA (Table 4). 95% confidence intervals for 2-back>0-back condition in GWI bracketed zero suggesting that BOLD may have been equivalent in the 2-back and 0-back trials. In contrast, control and CFS had greater BOLD activation during the high cognitive load 2-back working memory task compared to the low cognitive load 0-back attention task.

The model was significant for gender (Wilks' lambda = 0.798, p = 0.047, Partial Eta Squared = 0.202) (Table S15). Overall, males had greater BOLD activation than females after adjustment for the other variables, but the differences were only significant for R_LC and R_PBC.

Postexercise data were reevaluated by regression with BOLD values as the dependent variables, and age, gender, disease status, orthostatic status, PTSD status, BMI, dolorimetry thresholds (kg) as independent variables. The outcome (Table S16) restricted the number of significant regions to six (L_MRF, R_MRF, PAG, VTA, DR, R_PPN) and three variables.

This restricted model was significant for Disease status (Wilks' lambda = 0, p = 0.007, Partial Eta Squared = 0.981) after accounting for gender and dolorimetry (kg). Estimated marginal means (mean ± 95%CI) were significantly higher in control and CFS than GWI after exercise (univariate significance) (Table S17). The model did not reach significance for gender (Wilks' lambda = 0.161, p = 0.409, Partial Eta Squared = 0.839) but males had a trend for higher BOLD than females in PAG, VTA and R_PPN by univariate comparisons (Table S18).

The model was significant for dolorimetry thresholds (kg) (Wilks' lambda = 0, p = 0.049, Partial Eta Squared = 0.984) with significant univariate differences for PAG, VTA, DR and R_PPN (p < 0.042) (data not shown). However, average dolorimetry results were equivalent in male and female subgroups of control, CFS and GWI by ANOVA in this sample. This results was viewed with skepticism because a larger sample had shown significantly lower pain thresholds in GWI and CFS than control females [25].

Evaluations of postexercise data ended here to avoid overfitting the models.

Table S14. Disease status in postexercise mGLM. The mGLM was significant for Disease status postexercise (Wilks' lambda = 0.665, p = 0.033, Partial Eta Squared = 0.184). CFS and control had significantly higher BOLD activation than GWI.

	Control	CFS	GWI	Control>GWI	CFS>GWI
L_MRF	0.379 ± 0.218	0.237 ± 0.157	-0.045 ± 0.140	0.004	0.026
R_MRF	0.331 ± 0.206	0.348 ± 0.149	0.042 ± 0.132		0.008
PAG	0.204 ± 0.191	0.332 ± 0.137	-0.017 ± 0.122		0.001
VTA	0.249 ± 0.160	0.202 ± 0.115	-0.011 ± 0.102	0.022	0.021
DR	0.207 ± 0.177	0.310 ± 0.129	-0.016 ± 0.114		0.001
MR	0.193 ± 0.194	0.322 ± 0.140	-0.003 ± 0.125		0.002
L_PO	0.109 ± 0.210	0.256 ± 0.152	0.062 ± 0.135		

L_PPN	0.150 ± 0.203	0.250 ± 0.146	-0.022 ± 0.13		0.02
R_PO	0.179 ± 0.199	0.276 ± 0.145	0.017 ± 0.128		0.026
R_PPN	0.338 ± 0.182	0.295 ± 0.132	0.067 ± 0.117	0.042	0.033
L_LC	0.124 ± 0.228	0.290 ± 0.165	0.031 ± 0.147		
R_LC	0.133 ± 0.240	0.360 ± 0.173	-0.005 ± 0.154		0.007
L_PBC	0.171 ± 0.220	0.267 ± 0.159	0.023 ± 0.141		
R_PBC	0.147 ± 0.201	0.317 ± 0.146	0.032 ± 0.13		0.013

Table S15. Gender in postexercise mGLM. The model was significant for gender (Wilks' lambda = 0.798, p = 0.047, Partial Eta Squared = 0.202). Males had higher BOLD activation in R_LC and R_PBC than females.

	Male	95%CI	Female		Male>Female
L_MRF	0.206	0.127	0.114	0.144	
R_MRF	0.245	0.119	0.183	0.136	
PAG	0.186	0.11	0.111	0.126	
VTA	0.186	0.093	0.071	0.106	
DR	0.196	0.103	0.093	0.117	
MR	0.218	0.112	0.083	0.129	
L_PO	0.194	0.123	0.077	0.14	
L_PPN	0.156	0.118	0.061	0.134	
R_PO	0.191	0.116	0.091	0.133	
R_PPN	0.277	0.106	0.151	0.121	
L_LC	0.226	0.133	0.049	0.151	
R_LC	0.274	0.14	0.022	0.16	0.02
L_PBC	0.15	0.128	0.123	0.146	
R_PBC	0.255	0.117	0.052	0.134	0.026

Table S16. Postexercise regression analysis. Postexercise data were reevaluated by regression with BOLD values as the dependent variables, and age, gender, disease status, orthostatic status, PTSD status, BMI, dolorimetry thresholds (kg) as independent variables. The outcome restricted the number of significant nodes and variables to use in the second postexercise multivariate general linear model (Table S17).

	Significance for regression model	Univariate significance for Disease status	Univariate significance for dolorimetry	Univariate significance for gender
L_MRF	0.004	0	0.031	
R_MRF	0.003	0		
PAG	0.023	0.009	0.025	
VTA	0.002	0.001	0.017	0.014
DR	0.032	0.002		
MR	ns			

L_PO	ns			
R_PO	ns			
L_PPN	ns			
R_PPN	0.005	0.001	0.028	0.016
L_LC	ns			
R_LC	ns			
L_PBC	ns			
R_PBC	ns			

Table S17. Postexercise multivariate general linear model for Disease status based on regression analysis (Table S16). The model was significant for Disease status (Wilks' lambda = 0, p = 0.007, Partial Eta Squared = 0.981) after accounting for gender and dolorimetry (kg). Estimated marginal means (mean ± 95%CI) were significantly higher in control and CFS than GWI after exercise (univariate significance).

	Control	CFS	GWI	Control>GWI	CFS>GWI
L_MRF	0.341 ± 0.202	0.216 ± 0.191	0.065 ± 0.130		
R_MRF	0.364 ± 0.103	0.311 ± 0.098	0.066 ± 0.066	0.002	0.005
PAG	0.182 ± 0.087	0.280 ± 0.083	0.055 ± 0.056		0.003
VTA	0.252 ± 0.072	0.198 ± 0.068	0.078 ± 0.047	0.006	0.032
DR	0.229 ± 0.079	0.269 ± 0.074	0.063 ± 0.051	0.012	0.003
R_PPN	0.308 ± 0.080	0.291 ± 0.076	0.121 ± 0.052	0.007	0.01

Table S18. Postexercise multivariate general linear model for gender based on regression analysis (Table S14). Although the model was not significant for gender (Wilks' lambda = 0.161, p = 0.409, Partial Eta Squared = 0.839), males had greater BOLD than females by univariate significance for PAG, VTA and R_PPN.

	Male	Female	Males>Female
L_MRF	0.205 ± 0.120	0.096 ± 0.154	
R_MRF	0.217 ± 0.062	0.154 ± 0.079	
PAG	0.178 ± 0.052	0.072 ± 0.067	0.021
VTA	0.186 ± 0.042	0.080 ± 0.055	0.009
DR	0.178 ± 0.047	0.105 ± 0.060	
R_PPN	0.252 ± 0.048	0.126 ± 0.061	0.006

METHODS for Incremental changes (Postexercise minus Preexercise) by multivariate general linear models (mGLM) and Table S19.

mGLM for incremental changes in BOLD (ΔBOLD) were evaluated with Disease, Orthostatic, PTSD, gender as fixed factors and age, BMI and dolorimetry as independent variables.

RESULTS for Incremental changes (Postexercise minus Preexercise) by multivariate general linear models (mGLM) and Table S19.

The model was significant for Disease status (Wilks' Lambda = 0.664, p = 0.031, Partial Eta Squared = 0.185) and Orthostatic status (Wilks' Lambda = 0.651, p = 0.019, Partial Eta Squared = 0.193) but not age, gender, PTSD, BMI or dolorimetry.

Estimated marginal means for Disease status bracketed zero for controls, were positive for CFS and negative for GWI. Incremental changes were elevated in CFS and decreased in GWI for all nodes except the midbrain reticular formation (Table S19).

Table S19. Estimated marginal means for ΔBOLD and Disease status. Mean ± 95%CI. Univariate significance.

	Control	CFS	GWI	CFS > GWI
L_MRFΔ	0.052 ± 0.307	0.085 ± 0.222	-0.227 ± 0.198	
R_MRFΔ	-0.131 ± 0.312	0.080 ± 0.226	-0.086 ± 0.201	
PAGΔ	-0.052 ± 0.264	0.221 ± 0.191	-0.249 ± 0.170	0.001
VTAΔ	-0.065 ± 0.206	0.098 ± 0.148	-0.157 ± 0.132	0.035
DRΔ	-0.012 ± 0.245	0.190 ± 0.178	-0.283 ± 0.158	0.00036
MRΔ	-0.012 ± 0.264	0.178 ± 0.192	-0.317 ± 0.170	0.001
L_POΔ	-0.108 ± 0.267	0.200 ± 0.194	-0.209 ± 0.171	0.006
L_PPNΔ	-0.176 ± 0.250	0.232 ± 0.181	-0.308 ± 0.160	0.000059
R_POΔ	-0.027 ± 0.290	0.160 ± 0.211	-0.276 ± 0.187	0.007
R_PPNΔ	-0.046 ± 0.254	0.163 ± 0.184	-0.201 ± 0.164	0.011
L_LCΔ	-0.037 ± 0.306	0.089 ± 0.222	-0.290 ± 0.197	0.036
R_LCΔ	0.086 ± 0.381	0.192 ± 0.276	-0.376 ± 0.245	0.008
L_PBCΔ	-0.143 ± 0.295	0.090 ± 0.213	-0.294 ± 0.189	0.025
R_PBCΔ	-0.029 ± 0.308	0.187 ± 0.223	-0.249 ± 0.198	0.013
